# Effects of Dog-Assisted Therapies on Cognitive Mnemonic Capabilities in People Affected by Alzheimer’s Disease

**DOI:** 10.3390/ani11051366

**Published:** 2021-05-11

**Authors:** Fausto Quintavalla, Simona Cao, Diana Spinelli, Paolo Caffarra, Fiammetta M. Rossi, Giuseppina Basini, Alberto Sabbioni

**Affiliations:** 1 Dipartimento di Scienze Medico Veterinarie, Università di Parma, Via del Taglio 10, 43126 Parma, Italy; giuseppina.basini@unipr.it (G.B.); alberto.sabbioni@unipr.it (A.S.); 2 Società Cooperativa Sociale r.l. Killia, Via Svetonio 23, 09042 Monserrato, Italy; simona.cao@unipr.it (S.C.); psicologa@killia.it (D.S.); 3 Sezione di Neuroscienze, Dipartimento di Medicina e Chirurgia, Università di Parma, Via Gramsci 14, 43126 Parma, Italy; paolo.caffarra@unipr.it; 4 Associazione Jiva, Via Erberto Carboni, 2/D, 43123 Parma, Italy; piccolafiamma@hotmail.com

**Keywords:** Alzheimer’s disease (AD), animal-assisted interventions (AAIs), Mini-Mental State Exam (MMSE), Alzheimer’s Disease Assessment Scale (ADAS), Wellness and Cognitive Ability Questionnaire (Brief Assessment Cognition or BAC), senile dementia, elderly patients, dogs

## Abstract

**Simple Summary:**

Alzheimer’s disease is the most common cause of dementia in humans and, as the disease progresses, symptoms become more relevant, with significant interference in daily activities and social relations. Currently, a valid treatment is lacking and no highly effective drug has yet been approved for Alzheimer’s disease treatment. Animal-assisted interventions play a significant role in the lives of people with dementia. The purpose of the present study is to provide a contribution to research on elderly patients suffering from Alzheimer’s disease in whom dog-assisted therapies prove to be effective and fully validated during the period of time for which the patient has contact with the animal. The index of impairment of cognitive skills was assessed through different tests. Two months after the end of the sessions, the test results decreased to their initial values.

**Abstract:**

Alzheimer’s disease (AD) is the most common cause of dementia in humans and, currently, a valid treatment is lacking. Our goal is to demonstrate the importance and benefits of the relationship with companion animals (considered as co-therapists), intended as a means of facilitating social relations and promoting evident wellbeing in AD patients. The study involved 30 randomly chosen patients with Alzheimer’s disease (group T) and three dogs. The group participated in a total of 24 animal-assisted interventions (AAIs) sessions over a span of 12 weeks, using the Mini-Mental State Examination (MMSE), Wellness and Cognitive Ability Questionnaire (Brief Assessment Cognition or BAC), and Alzheimer’s Disease Assessment Scale (ADAS) as assessment tests. A second group (group C), consisting of 10 people with AD, was enrolled as control group and underwent the same assessment tests but did not benefit from the presence of the dogs. Tests were carried out at time T0 (before starting sessions), T1 (end of sessions), and T2 (two months after last session). People belonging to group T achieved an overall improvement in their perceived state of wellbeing, even on a cognitive and mnemonic plane. However, two months after the end of the sessions, the test results in people suffering from AD decreased towards the baseline (T0). The study shows how such progress can be achieved through activities based on the relationship with an animal, as long as the animal is a steady presence in the life of the patient receiving the intervention. Dogs involved in other dog-assisted therapies have been found suitable also for assisting patients with AD.

## 1. Introduction

Alzheimer’s disease (AD) is the most common cause of human dementia. According to some surveys, a new person is affected by dementia every 3 s [1]. The Italian organization Alz.org^®^ reported in 2019 that more than 1 million people in Italy were living with dementia. It has been estimated that, in 2020, 584,000 new cases of dementia would occur in the country. In addition to affecting memory and mental functions (e.g., thinking, language skills, orientation, etc.), behavioral and psychological symptoms (walking, sleeping, or sexual problems) are also present. Multiple risk factors have been identified, including female gender, age, low education level, the apolipoprotein E (APOE * E4 allele), smoking, obesity, and diabetes mellitus [2]. Dementia is preceded by an intermediate condition termed mild cognitive impairment (MCI), characterized by normal activity on a daily basis. As the disease progresses, symptoms become more severe, affecting also the caregivers [3]. Behavioral disorders may also be present (aggression, reluctance, wandering, anxiety, crying), increasing the burden of daily management. No highly effective drug has yet been approved for treating AD [4]; nevertheless, today, the treatment of AD patients is multidisciplinary, widely based on psychological and rehabilitative approaches to delay the progressive loss of function and to maintain patients’ residual cognitive abilities and quality of life [5].

Complementary therapies are becoming more popular in integrating traditional drug therapies, and non-pharmacological treatment should always be considered first. These integrative approaches are non-invasive, with minimal side effects, and are definitely less costly. The most used are “Doll Therapy”, which emerged in the 1980s in the USA and Australia [6]; art therapy and music therapy, which aim to enrich the lives of people affected by dementia; and writing therapy, with which patients may remember long-past memories, recall and process past emotions, and induce a deeper self-talk [7]. Among these integrative therapies, animal-assisted interventions (AAIs) play a major role [8,9,10]. The IAHAIO (International Association of Human-Animal Interaction Organizations) White Paper 2014 (IAHAIO.org) defined AAIs as “*a goal oriented and structured intervention that intentionally includes or incorporates animals in health, education and human services for the purpose of therapeutic gains in humans*”. Through physical, emotional, social, or cognitive functions in both healthy and unhealthy individual or group interventions, they contribute to the improvement of people’s wellbeing. It has long been known that companion animals play an important part in the lives of people of different ages and backgrounds. Humans and pet dogs respond to non-verbal interactions with a decrease in blood pressure and an increase in neurochemical substances associated with relaxation and bonding [11] and other hormones such as oxytocin, prolactin, and dopamine. With regard to treating AD, there are several case studies available in the scientific literature involving both real dogs as well as robotic pets [7,11,12,13,14]. The AAIs focus mainly on improving the quality of life, especially in elderly people, through educational, re-educational, and/or recreational activities carried out by specialized operators and/or volunteers, with the help of companion animals (co-therapist pets) selected according to specific requirements [3,7,11,13,15,16,17,18,19]. AAIs represent a very complex system of relational feedbacks based on physical gestures and attitudes, as well as activation of emotional sense–motor models between the two different species involved. Based on the scientific literature, different animal species (dogs, horses, cats, fish, canaries) are involved in AAIs but it seems that dogs, more than others, help to build a relationship based on greater reciprocity with deeper therapeutic effects [10,20,21]. Moreover, due to ethological characteristics, dogs not only learn through play, just like children, but are prone to establishing active relationships, communications, and interactions [22]. In recent years, several studies have been conducted on the effectiveness of dog-assisted therapies in AD patients [10,23,24,25,26,27], but some studies concluded that the improvements observed were not always significant [28,29,30].

The purpose of the present study is to provide a contribution on the fundamental role of the human–animal relationship in elderly patients suffering from AD, involving dogs with a role in dog-assisted therapies. In particular, the aims of the research were: (i) to establish rewarding emotional relationships between patients and dogs through caring activities, play, affectivity, psychomotor activities, cognitive activities; (ii) to promote concentration abilities in patients, involving dogs to recall memories of the past and promote patients’ willingness to share them in a group; (iii) to stimulate patients’ motor skills through simple, fine, and general mobility exercises (e.g., throwing a ball to fetch, brushing the dog’s coat, etc.); (iv) to promote patients’ self-confidence and self-esteem through building an influential relationship with an animal; (v) to break the monotony of scheduled routines with different and enjoyable activities; (vi) to assess the influence of environmental factors (gender, age, cultural background) on behavioral parameters.

## 2. Materials and Methods

### 2.1. Population Study

Forty patients diagnosed with AD and hosted at day-care center “Don Orione” in Selargius (Sardinia, Italy) were selected on the basis of the following criteria:

- medium to severe degree of impairment with an MMSE (Mini-Mental State Examination) score of at least 4;
- no aversion, rejection, or fear of dogs;
- (as reported by day-care staff) minimal mnemonic skills that allow recall of previous sessions with the dog;
- minimal ability to communicate personal feelings;
- overall difficulties in taking part in social activities;
- need of a one-to-one relationship.

Out of these, 30 people were randomly selected (group T) and then later divided into 10 subgroups, each consisting of 3 randomly chosen people of mixed age, gender, and schooling level (Table 1). Both experimental as well as control groups were randomly made up of patients suffering from AD.

Each subgroup of group T attended 24 AAI sessions, always with the same co-therapist dog; sessions occurred twice a week in the morning and lasted 30 min each, over a 12-week period. At the same time, a control group (group C) was created, consisting of 10 people who did not participate in the AAI sessions. Aside from these sessions, both groups C and T performed the same other activities and followed scheduled routines offered by the day-care center; the only change was involving 30 people in group T in the AAI sessions. All patients and caregivers, when necessary, gave their informed consent to participate in this study, according to good clinical practices. This study plan was submitted to the Committee for Animal Ethics of the University of Parma, Italy (approval number 08B-CE20), and tests were conducted in accordance with approved guidelines.

### 2.2. Animals

Three dogs were involved in this project, all owned by AAIs operators working for the Killia Cooperative:

- “Giulia”, Staffordshire Bull Terrier, sterilized female, 7 years old, 17 kg;
- “Nala”, Labrador retriever, female, 2 years old, 30 kg;
- “Mia”, Mongrel, sterilized female, 5 years old, 8 kg.

The approach used in educating dogs is known as a cognitive zooanthropological (CZ) approach. It focuses on the mental aspects of animals, stating that their behavior is not the result of automatic reflexes but of each individual’s subjective mental processes instead, and it can be influenced by social and environmental surroundings [31]. Animals are considered as bearers of unique features and seeing the world through their eyes can promote empathy in humans, leading them towards wider perspectives and a deeper knowledge of themselves. On a practical level, this approach focuses on deeply understanding and responding effectively to animals’ basic ethological needs, thus building a relationship of mutual trust and respect, promoting appropriate contact with companion animals, and improving their integration in social and family environments.

The dogs had been regularly followed from a behavioral point of view following precise protocols based on dog training programs, and they were certified suitable for AAIs. They were all living in comfortable and respectful family environments, according to values and principles of the Italian declaration on relations with pets [32,33]. Eligibility as co-therapist dogs is reassessed annually by a veterinarian expert in animal behavior and AAIs, as well as by dog trainers and instructors.

From a sanitary standpoint, all enrolled animals received regular vaccination shots against rabies, distemper, parvovirus, canine hepatitis, leptospirosis, as well as treatments to prevent heartworm disease and infestations of other internal/external parasites (fleas, ticks, sandflies). Dogs also underwent annual physical examinations that included blood biochemical-clinical tests.

At the preliminary stage, at midpoint, and at the end of the project, each co-therapist dog underwent a behavior assessment by a veterinarian expert in animal behavior and AAIs. Furthermore, AAI operators received monitoring forms which were to be filled in at the end of each session, in order to keep dogs’ conditions and wellbeing constantly monitored. Monitoring animal welfare is not only compulsory for AAIs in our country but also represents a key factor in our study since our approach implies a strong bond between operators and pets involved, thus respecting pets’ physical and mental wellbeing at all times (Figures S1 and S2).

### 2.3. Setting

Before starting the research, the environment was accurately considered to define a suitable setting for testing the patients involved and conducting AAI sessions with pets. A wide room with few pieces of furniture was chosen, in order to have enough useful space to perform all planned activities, with two separate access points so patients and dogs could move more independently. Furthermore, an area in the room was designated for the co-therapist dog to rest and relax, where a dog blanket and bowl of water were placed. In this setting, three chairs were placed in the middle of the room to accommodate patients, at a distance of approximately 1 m apart so as to respect personal space. The ambience met the requirements for both dogs and patients in creating a communicative shared experience.

### 2.4. Project Outline

The project complied with the Italian National Legislation (Rep. Acts no. 60 CSR, 25 March 2015) and resolution no. 15/12, 21 March 2017 of the Sardinia Region regarding AAIs.

Proposed activities were planned so that each week had a well-defined topic with two sessions focused on the same theme/activity, for a total of 12 different activities (Table 2).

Each subgroup of group T attended 24 AAI sessions, always with the same co-therapist dog; sessions occurred twice a week in the morning and lasted 30 min each, over a 12-week period. At the same time, a control group (group C) was created, consisting of 10 persons who did not participate in the AAI sessions. Aside from these sessions, both groups C and T performed the same other activities and followed scheduled routines offered by the day-care center; the only change was involving 30 persons in group T in the AAI sessions.

### 2.5. Performed Tests

The Mini-Mental State Examination (MMSE), the Questionnaire of Wellness and Cognitive Abilities (Brief Assessment Cognition or BAC), and Alzheimer’s Disease Assessment Scale (ADAS) [34,35] served as measures of cognitive functions at baseline (T0), T1 (end of session), and T1 (2 months after the last session) for each group.

Administration of the MMSE test takes around 10–15 min. It consists of 11 items assessing: temporal orientation, spatial orientation, immediate memory (fixation or recording memory), attention and calculation, memory, language—denomination, language—repetition, language—oral comprehension, language—reading and written comprehension, language—generation of a written sentence, copy of drawing (constructive praxis). However, cut-off scores may vary greatly, since factors such as age and schooling level significantly contribute to variations in scores expected in the normal population; for this reason, adjustment coefficients were developed. The total score ranges from a minimum of 0 (very severe) to a maximum of 30. The global score was then adjusted for age and schooling as reported by Magni et al. [36]. An adjusted score higher than 24 is considered normal.

The BEN-SSC questionnaire, part of the Wellness and Cognitive Abilities (BAC) portfolio in adulthood and advanced age, was used for assessing general wellbeing. The BEN-SSC questionnaire is a tool that measures wellbeing as perceived by a subject. The questionnaire consists of 37 items in a positive form (without negations) and uses subtests to measure:

- personal satisfaction (BENSP): related to one’s past (all that has been achieved in life, despite challenges) and present life (liking oneself, being satisfied with present and future expectations);
- coping strategies (BENSC), intended as the ability to face minor and major daily issues, perceiving one’s ability of “knowing how to do” something;
- sense of autonomy and independence;
- emotional skills (BENCE), intended as the ability to recognize and understand one’s own emotions and those of others, satisfaction in social relationships;
- finally, a score for global overall wellbeing (Total Wellbeing).

In the BAC test, obtained scores must be adjusted according to age, gender, and schooling level.

After presenting each item, the patient is asked to consider how he/she perceives the statement according to a 4-point Likert scale (from “Never” = 1 to “Always” = 4). All relative scores for different skills (BENSP, BENSC, BENCE) are used to calculate the Total Wellbeing score. All items of each single subtest are added up and a single final value is obtained.

To convert the raw score, it is necessary to use scoring software that requires the input of age range, schooling level, and scores on the Likert scale for each item.

Performing the ADAS Test requires around 30–40 min, prior to which a short conversation with the patient takes place, focusing on neutral topics such as weather, patient’s breakfast, etc. (ADAS-Cog). It consists of 11 tests designed to assess short- and medium-term memory (recalling words; recognition of words; learning instructions of a test); temporal–spatial orientation; language (verbal ability, difficulty in naming in spontaneous language, understanding of spoken language, naming of objects and fingers, execution of commands); aphasia; attention and concentration. Scores range from 0 (no impairment) to 70 (very severe impairment). In this test, obtained scores must be adjusted according to schooling level with the of a specific grid. Scores of 0–30 refer to patients with mild deterioration and mental impairment ranging from absent to third degree scores; scores of 31–51 refer to patients with moderate deterioration and mental impairment ranging from fourth to eighth degree; scores of 52–70 refer to patients with severe deterioration and mental impairment ranging from ninth to thirteenth degree.

It is important to keep in mind that in the ADAS test, a higher score means higher impairment, while in the MMSE test, a higher score means better performance/abilities.

Each session was organized so that patients would enter the room, with the operators and co-therapist dogs already being there. This way, the dogs would welcome and greet the elderly patients. A routine was devised so that all sessions started and ended with operators and dogs greeting each individual patient in turn, creating thus a direct patient–dog relationship. Each patient group was paired up randomly with a co-therapist dog, which remained with that group throughout all 24 sessions.

Each proposed activity implied the active participation of all patients, with possible spontaneous participation as a group.

### 2.6. Data Analysis

The data collected in this study were processed with analysis of variance (IBM SPSS Statistics, ver. 26.0.0.1, 2020) according to a model containing the group fixed factors (2 levels: experiment, control), patients’ age range (4 levels: 60–69; 70–79; 80–89; 90+ years old), patients’ gender (2 levels: male, female), schooling level (4 levels: up to 5 years; up to 8 years; 8/13 years; 13+ years), timings (3 levels: T0; T1; T2), co-therapist dogs (4 levels: Giulia; Mia; Nala; no dog), first-level interactions of group with time, age, gender, schooling level, and second-level interactions among group, gender, time. The differences between the estimated means were considered significant when *p* < 0.05; when 0.05 < *p* < 0.10, the differences were considered as a tendency.

## 3. Results

The age range of enrolled patients was between 63 and 98 years old. The female gender was over-represented in comparison to males (29 vs. 11).

The effects of time on groups revealed differences only in group T for all evaluated parameters (*p* < 0.05), except for Global Cognitive Evaluation ADAS (*p* > 0.05). Moreover, there were constant improvements in group T from time T0 to T1, with values decreasing over time in T2 for the MMSE test and Skills Emotional BAC, while values remained unchanged in other BAC subtests (Table 3). Differences between groups were negligible and limited by time, referring to MMSE and ADAS tests at T0, Total Wellbeing BAC, Coping Strategies BAC at T1 and T2, and Emotional Skills BAC at T1 (*p* ≤ 0.05).

In particular, variance analysis revealed a significant effect (*p* < 0.05) of group and age on all tests carried out; presence of dogs (within groups) was a significant source of variability for all tests except on Emotional Skills (BAC), while schooling level influenced all tests, except Global Cognitive Evaluation (MMSE and ADAS). Other main model factors (gender and time) never reached statistical significance (*p* > 0.05). Interactions’ effects were characterized by certain variability with respect to the various tests carried out, with a clear prevalence of significant effects on Total Wellbeing (BAC), Coping Strategies (BAC), and Emotional Skills (BAC). The adopted model explained percentages of variability ranging between 42.0% for Global Cognitive Evaluation (ADAS) and 58.1% for Total Wellbeing (BAC-SSC).

Regarding time, females in group T showed an improvement from 15.78 to 19.38 (*p* < 0.05) in the MMSE test; in the ADAS test, they worsened from 28.84 to 23.89 (non-significant difference); in the BAC Questionnaire, the difference between T0 and T1 was significant, while in men, it represented only a tendency.

Regarding schooling level, differences between the two groups were significant (*p* < 0.05) and in favor of group T in those with schooling up to 8 years in Total Wellbeing BAC, Personal Satisfaction BAC, and Coping Strategies BAC, while in Emotional Skills BAC, significant differences, in favor of group T, were highlighted only for schooling up to 5 years. For Global Cognitive Evaluation MMSE, differences between the two groups were in favor of group C, with schooling level between 5 and 8 years. No significant differences emerged for higher levels of education, although the sample showed a lack of highly educated subjects in the control group (Table 4).

Animal therapy affected the quality of life of AD patients in terms of both perceived wellbeing and on cognitive and mnemonic levels, in comparison to control group C (Table 5).

Questionnaires on physical and mental assets of pets involved, filled out by operators at the end of each session, highlighted a constant and unchanged state of global animal welfare throughout the entire period of time, without ever showing signs of discomfort or stress.

## 4. Discussion

Dementias are considered to be an expression of an organic cerebral disease of degenerative, vascular, or mixed nature, which influences cognitive skills, daily activities, and behavior. Alzheimer’s disease (AD) is an irreversible, mostly sporadic, degenerative condition, with age of onset usually after age 65, but may occur earlier as in families with genetic transmission [3]. We recruited subjects mostly aged >80, mostly females, with an average schooling level similar to populations described by other authors [2,37].

AAIs evokes memories, and there is a sense of physical closeness during the time that the person spends stroking the dog. However, interaction with a therapy dog can also evoke sadness, which was not reported in our sample but which may be difficult for people to handle [38].

Three dogs were involved in this project having different anamnestic history as well as different breed profiles. Breeds and age were found to be a significant source of variability in 5 out of 6 parameters, and the presence of only three different breeds/sizes/ages is not sufficient to express an opinion about the best combination of the above. The main factors in selecting animals are not related to specific cognitive skills nor to morphology, but mostly to their relational and bonding characteristics.

Dogs’ cognitive abilities had effects on patients with AD, which were evaluated through MMSE, ADAS-Cog, and BAC. Of the three tests used, only the first was used by other authors in AAI studies. The MMSE test is a fast and sensitive tool for exploring cognitive functions and their changes over time in people suffering from AD; it is also applicable in severe forms of deterioration [39]. Our data are similar to those reported by other authors [15,18,37,40], showing an increase in MMSE score at time T1 in group T compared to time T0, similar in women and men (3.6 points and 4 points, respectively). By contrast, group C shows a decrease of 1.34 and 4 points, respectively, in women and in men. Motomura et al. [28] showed no significant differences before and after dog therapy. Experiences related to benefits of human–animal relation assessed with the BAC questionnaire are scarce. BAC tests include subtests that provide a measure of passive and active short-term memory capabilities, as well as verbal memory capabilities. Antonelli and Cusinato [41] reported that physical contact with a dog stimulated female participants to recall memories and share them with the rest of the group.

In our study, both men and women, randomly, were delighted to tell us about their past experiences with pets and how their habits and ways of living with dogs have changed over the years.

The ADAS-Cog is a reliable and valid tool that has become a standard outcome measure in multi-national AD treatment trials [42,43,44]. While not being a substitute for extensive neuropsychological assessment, ADAS-Cog is considered more complete than most other cognitive screening measures, and in previous studies was uninfluenced by age, gender, or schooling level (except within low education range of 0–6 years) [45]. However, in our case, it was influenced by age.

Improvements in social behavior have been found to be unrelated to severity of dementia [13]. This result also emerged from our research as many patients improved their social behavior and perceived global wellbeing.

No study adopted a randomized controlled trial design, and a number of potentially important factors were not controlled, including effects of animals on caregivers that may bias caregivers’ responses when acting as proxies for their relatives or residents [13].

Two months after the end of sessions, in phase T2, test results decreased to the baseline (T0), suggesting that the benefits of the dog interventions are limited to the active phase and should not discontinued for long time intervals. Future studies are needed to further explain the beneficial effects of dog therapy approaches for patients with dementia in order to consolidate positive results.

Our work has some limitations, mainly identified as using small subject samples; for some categories of patients, AAIs should be tailored to their to their specific needs and interests, aiming at patient-centered dementia care. Since AD is a degenerative disease, a possible solution could be to plan a higher number of dog therapy sessions in a shorter span of time in order to limit the progression of the disease itself. Moreover, it would be interesting to observe if, after the follow-up period, patients return to their previous cognitive mnemonic capabilities. We are also aware that a further limitation of this study is the low number of animals involved. The choice of dogs was based not only on hereditary phylogenetic aspects of breeds but mainly on acquired ontogenetic characteristics, with a veterinarian expert in animal behavior and AAIs evaluating each dog–operator couple at the present moment, “here and now” (considering present social, environmental, situational context).

## 5. Conclusions

Our hypothesis is validated since employing AAIs in patients suffering from AD is fully justified for the period of time during which a patient has contact with an animal. Over a two-month period (T2), the gained results reverted to their initial time (T0) values, proving how the animal’s presence is beneficial when constant in a patient’s life.

AAIs contribute to the improvement of social behavior. The presence of a dog stimulates patients with AD to interact and thus reduces their social isolation and loneliness. Employing AAIs in patients suffering from AD is fully justified only for the period of time during which a patient has contact with an animal. Dogs, regardless of size and breed, are suitable for this type of activity with patients affected by AD. With regard to priorities in identifying suitable dogs, it would be necessary to involve co-therapist dogs in activities, focused on each individual’s specific characteristics regarding high levels of interspecific social and collaborative motivations.

Finally, there must be a secure dog–operator attachment bond.

Moreover, future research on AAIs in elderly patients with dementia should also include assessments in family members and caregiving staff in order to have a wider overview of complex interactions among members involved in such paradigms.

## Figures and Tables

**Table 1:** AD patients divided according to age, schooling level, and gender in experimental group (group T) and control (group C).

Experimental Group (T Group)				Control Group (C Group)			
Parameters	Category	N° Patients	%	Parameters	Category	N° Patients	%
Age	60+	4	13.3	Age	60+	2	20
	70+	10	33.3		70+	3	30
	80+	11	36.7		80+	4	40
	90+	5	16.7		90+	1	10
Schooling Level	Up to 5 years	14	46.7	Schooling Level	Up to 5 years	6	60
	Up to 8 years	3	10.0		Up to 8 years	3	30
	8/13 years	11	36.7		8/13 years	1	10
	Over 13 years	2	6.7		Over 13 years	0	0
Gender	Male	8	26.7	Gender	Male	3	30
	Female	22	73.3		Female	7	70
Mortality during procedure		3	10	Mortality during procedure		1	10

**Table 2:** Scheduled sessions, proposed activities, and goals.

Week	Proposed Activities	Means	Aims
Week 1	Introducing the dog (its features), talk, practical demonstration	Storytelling and relationships within the group; talking about oneself and what he/she can do well	Patients and dog get to know each other; long-term memory
Week 2	Introducing the dog (what dog is unable to do well or has not learned yet), talk, practical demonstration	Talking about oneself; understanding that everyone has strengths and weaknesses	Patients and dog get to know each other; long-term memory and coherent narrating
Week 3	Nose work (olfactory search) with objects (different colors, shapes, front/back, big/small)	Work on dog’s senses	Observation and description of events; hypothetical thinking; discriminating between different characteristics; long-term memory
Week 4	Solitaire dog games (industrial, cardboard-made)	Play as a medium	Recalling games when they were young; entertaining with dog’s games and functions, watching it in action; long-term memory; observation and description skills
Week 5	Searching images (dice, animals, bowls)	Recognizing animal images and pictures on bowls and on sides of dice	Discriminating images; specific work on dysnomia
Week 6	Image search and recognition	Recognizing images of everyday objects, depicted on laminated cards	Work on dysnomia and short-term memory
Week 7	Home-made and industrial feeding: dogs too have preferences	Recognizing food through smell/sight; repeating names of foods seen	Sensory stimulation; stimulating sensory memory (smell and sight)
Week 8	Care and massage	Enjoying spontaneous moments relaxing through grooming the dogs	Caring for other; opening to spontaneous memories; relaxing
Week 9	Doggy brain train	Industrial and home-made cognitive games for dogs	Specific problem-solving; hypothetical and observation thinking skills
Week 10	Standard mobility path	Promoting self-effectiveness in knowing “how to do”	Global mobility, coordination, and harmonious relationship with dog
Week 11	Home-made mobility path	Self-effectiveness in knowing “how to do”	Global mobility, coordination, and relationship in tune with dog; recognition of daily objects
Week 12	Recalling favorite activity mostly appreciated by the single group	Remembering activities carried out together and choosing favorite one	Promoting use of short- and long-term memory

**Table 3:** Effects of time on cognitive and behavioral variables (least squares means ±SE).

Parameter	Time	Control (Group C)	±SE	Experimental (Group T)	±SE
Global Cognitive Evaluation MMSE	T0	21.94 ^b^	2.02	14.35 ^aA^	1.39
	T1	19.38		18.48 ^B^	
	T2	17.72		15.46 ^A^	
Global Cognitive Evaluation ADAS	T0	16.98 ^a^	4.77	33.36 ^b^	3.27
	T1	20.18		28.36	
	T2	23.00 ^c^		32.50 ^d^	
Total Wellbeing BAC	T0	94.95	4.68	96.06 ^A^	3.21
	T1	91.06 ^a^		109.17 ^bB^	
	T2	90.62 ^a^		103.54 ^bB^	
Personal Satisfaction BAC	T0	31.75	1.87	33.35 ^A^	1.28
	T1	30.97 ^a^		36.65 ^bB^	
	T2	29.86 ^a^		34.91 ^bAB^	
Coping Strategies BAC	T0	19.68	1.49	19.43 ^A^	1.02
	T1	16.68 ^a^		23.58 ^bB^	
	T2	18.46 ^a^		22.28 ^bB^	
Skills Emotional BAC	T0	24.60	1.56	26.15 ^A^	1.07
	T1	24.93 ^a^		29.04 ^bB^	
	T2	24.60		26.89 ^A^	

^a,b^: significant difference (*p* < 0.05) between groups within time; ^c,d^: difference (*p* < 0.10) between groups within time considered as tendency; ^A,B^: significant difference (*p* < 0.05) between times within group.

**Table 4:** Estimated averages (±SE) of interaction between schooling level and group.

Schooling Level	Group	Global Cognitive Evaluation MMSE	Global Cognitive Evaluation ADAS	Total Wellbeing BAC	Personal Satisfaction BAC	Coping Strategies BAC	Emotional Skills BAC
Up to 5 years	Control	15.52 ± 1.92	27.94 ± 4.54	73.86 ± 4.46 ^a^	24.56 ± 1.78 ^a^	15.74 ± 1.42 ^a^	19.58 ± 1.48 ^a^
	Experimental	16.44 ± 1.30	26.36 ± 3.06	105.01 ± 3.00 ^b^	35.23 ± 1.20 ^b^	21.14 ± 0.96 ^b^	27.79 ± 1.00 ^b^
Up to 8 years	Control	20.26 ± 1.83 ^b^	17.96 ± 4.30	98.39 ± 4.23 ^a^	31.34 ± 1.69 ^a^	19.54 ± 1.35 ^a^	28.78 ± 1.41
	Experimental	15.03 ± 2.24 ^a^	25.70 ± 5.27	113.07 ± 5.18 ^b^	38.28 ± 2.07 ^b^	25.59 ± 1.65 ^b^	28.60 ± 1.72
8/13 years	Control	23.26 ± 4.03	14.26 ± 9.50	104.39 ± 9.34	36.68 ± 3.73	19.54 ± 2.98	25.78 ± 3.11
	Experimental	17.77 ± 1.48	26.12 ± 3.48	104.56 ± 3.41	32.42 ± 1.36	22.38 ± 1.09	28.57 ± 1.14
Over 13 years	Control	-	-	-	-	-	-
	Experimental	15.15 ± 3.99	47.46 ± 9.39	89.05 ± 9.23	33.94 ± 3.69	17.95 ± 2.95	24.48 ± 3.07

^a,b^ significantly different for *p* < 0.05.

**Table 5:** Group effect (presence/absence of dog) on cognitive and behavioral variables (least squares means ± SE).

Benchmark/Parameter	Control	Experimental
Global Cognitive Evaluation MMSE	19.68 ± 1.39 ^b^	16.10 ± 1.10 ^a^
Global Cognitive Evaluation ADAS	20.05 ± 3.28 ^a^	31.41 ± 2.59 ^b^
Total Wellbeing BAC	92.21 ± 3.22 ^a^	102.92 ± 2.54 ^b^
Personal Satisfaction BAC	30.86 ± 1.29 ^a^	34.97 ± 1.02 ^b^
Coping Strategies BAC	18.28 ± 1.03 ^a^	21.76 ± 0.81 ^b^
Emotional Skills BAC	24.71 ± 1.07 ^a^	27.36 ± 0.85 ^b^

^a,b^ different for *p* < 0.05.

## Data Availability

Data will be available on request.

## Supplementary Materials

The following are available online at https://www.mdpi.com/article/10.3390/ani11051366/s1, Figure S1: Dog monitoring forms used by AAI operators, with relative scores. The survey was responded to using a 5-point Likert frequency scale, answering “*never*”, “*rarely*”, “*sometimes*”, “*frequently*”, and “*very often*”, Figure S2: Monitoring dogs’ welfare. On the vertical axis the dog wellbeing is represented on a 0–5 range score. On the horizontal axis are numbers of questions answered throughout a total of 24 sessions.
