# Supplementary material for: Effects of Dog-Assisted Therapies on Cognitive Mnemonic Capabilities in People Affected by Alzheimer’s Disease
Source: Animals (Basel). 2021 May 11;11(5):1366. doi: 10.3390/ani11051366 (PMC8151255; doi:10.3390/ani11051366)
Supplement: Supplementary file 1 [file animals-11-01366-s001.zip › animals-1169143- supplementary-for the publication-11-5.pdf]

animals

Supplementary Materials

Effects of Dog-Assisted Therapies on Cognitive Mnemonic Capabilities in People Affected by Alzheimer's Disease

Fausto Quintavalla [1,*], Simona Cao [2], Diana Spinelli [2], Paolo Caffarra [3], Fiammetta M. Rossi [4], Giuseppina Basini [1] and Alberto Sabbioni [1]

1 Dipartimento di Scienze Medico Veterinarie, Università di Parma, Via del Taglio 10, 43126 Parma, Italy; giuseppina.basini@unipr.it (G.B.); alberto.sabbioni@unipr.it (A.S.)
2 Società Cooperativa Sociale r.l. Killia, Via Svetonio 23, 09042 Monserrato, Cagliari, Italy; simona.cao@unipr.it (S.C.); psicologa@killia.it (D.S.)
3 Sezione di Neuroscienze, Dipartimento di Medicina e Chirurgia, Università di Parma, via Gramsci 14, 43126 Parma, Italy; paolo.caffarra@unipr.it
4 Associazione Jiva, Via Erberto Carboni, 2/D, 43123 Parma, Italy; piccolafiamma@hotmail.com
* Correspondence: fausto.quintavalla@unipr.it; fax: +39-0521-032692

Citation: Quintavalla, F.; Cao, S.; Spinelli, D.; Caffarra, P.; Rossi, F.M.; Basini, G.; Sabbioni, A. Effects of Dog-Assisted Therapies on Cognitive Mnemonic Capabilities in People Affected by Alzheimer's Disease. *Animals* **2021**, *11*, 1366. https://doi.org/10.3390/ani11051366

Academic Editor: Serenella D'Ingeo, Angelo Quaranta and Marcello Siniscalchi

Received: 18 March 2021
Accepted: 4 May 2021
Published: 11 May 2021

Publisher's Note: MDPI stays neutral with regard to jurisdictional claims in published maps and institutional affiliations.

Copyright: © 2021 by the authors. Submitted for possible open access publication under the terms and conditions of the Creative Commons Attribution (CC BY) license (http://creativecommons.org/licenses/by/4.0/).

	QUESTIONS
1	The dog showed stress signals during the activities.
2	The dog showed calming signals during the activities.
3	The dog is happy while entering in the AAE building/area.
4	The dog looked for human partner's help during critical moments.
5	The dog took distance from human partner during critical moments.
6	The dog got physical injuries during the activities.
7	The dog suffered psychological or emotional traumas during the activities.
8	The dog has been interested in the activities.
9	The dog refused to interact with the patients.
10	The dog proposed spontaneous interaction with the patients.
11	The dog showed a proactive attitude.
12	The dog stayed close to the patient and to the human partner during the activities.
13	The dog showed pleasure in interacting with people during the activity.
14	The dog slept longer than usual after the activities.
15	The dog drank more water than usual during the activities.
16	The dog drank more water than usual at the end of the activities.

Figure S1. Dog monitoring forms used by AAI operators, with relative scores. The survey was responded to using a 5-point Likert frequency scale, answering "*never*", "*rarely*", "*sometimes*", "*frequently*", and "*very often*".

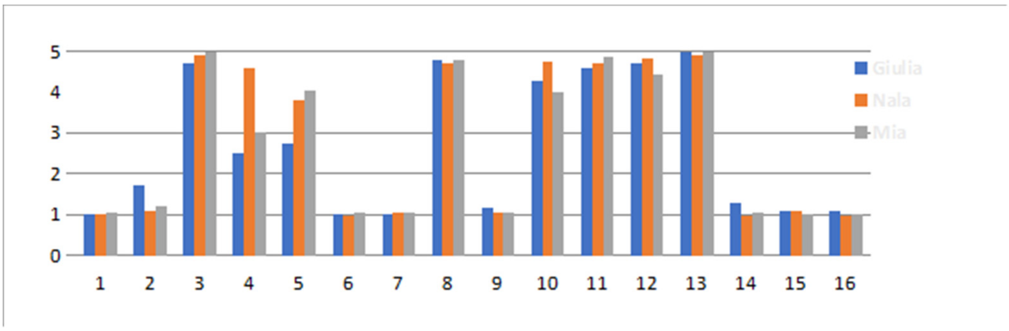

Figure S2. Monitoring dogs' welfare. On the vertical axis the dog wellbeing is represented on a 0–5 range score. On the horizontal axis are numbers of questions answered throughout a total of 24 sessions.
